# Effect of antiepileptic drugs in glioma patients on self-reported depression, anxiety, and cognitive complaints

**DOI:** 10.1007/s11060-021-03747-1

**Published:** 2021-04-06

**Authors:** Pim B. van der Meer, Johan A. F. Koekkoek, Martin J. van den Bent, Linda Dirven, Martin J. B. Taphoorn

**Affiliations:** 1 Department of Neurology, Leiden University Medical Center, PO BOX 9600, Leiden, 2300 RC The Netherlands; 2 Department of Neurology, Haaglanden Medical Center, The Hague, The Netherlands; 3 Department of Neurology, Erasmus Medical Center, Rotterdam, The Netherlands

**Keywords:** Anticonvulsants, Seizures, Glioma, Depression, Anxiety, Cognition

## Abstract

**Introduction:**

AEDs have been associated with depression, anxiety, and cognitive impairment, all frequent complications of glioma and its subsequent treatment, with considerable morbidity and an adverse effect on health-related quality of life. This study aimed to determine the independent association between AED use and self-reported depression, anxiety, and subjective cognitive impairment in glioma patients.

**Methods:**

In this multicenter cross-sectional study, depression and anxiety were assessed with the HADS and subjective cognitive impairment was assessed with the MOS-CFS. Univariable logistic regression analyses were performed on all potential confounding predictor variables. Potential confounders were included in the multivariable analyses if *p*-value < 0.1, to evaluate whether use of AEDs was independently related to depression, anxiety, and/or subjective cognitive impairment.

**Results:**

A total of 272 patients were included. Prevalence of depression differed significantly between patients not using (10%) and using AEDs (21%, unadjusted Odds Ratio [uOR] = 2.29 [95%CI 1.05–4.97], *p* = 0.037), but after correction for confounders the statistical significant difference was no longer apparent (adjusted Odds Ratio [aOR] = 1.94 [95%CI 0.83–4.50], *p* = 0.125). Prevalences of anxiety (aOR = 1.17 [95%CI 0.59–2.29], *p* = 0.659) and subjective cognitive impairment (aOR = 0.83 [95%CI 0.34–2.04], *p* = 0.684) did not differ significantly before or after adjustment of confounders between patients not using (19% and 16%, respectively) and using AEDs (26% and 21%, respectively).

**Conclusions:**

Our results indicate AED use was not independently associated with concurrent depression, anxiety, or subjective cognitive impairment in glioma patients. Alternative factors seem to have a greater contribution to the risk of developing neuropsychiatric symptoms in glioma patients.

**Supplementary Information:**

The online version contains supplementary material available at 10.1007/s11060-021-03747-1.

## Introduction

Gliomas account for almost 80% of all primary malignant brain tumours [1]. Patients with a glioma may face a variety of symptoms including mood disorders, cognitive dysfunction, and seizures [2–5]. Between 30% and 85% of patients with grade II–IV glioma experience epileptic seizures during the course of their disease [6, 7]. Subjective cognitive impairment (80%, assessed by reviewing medical records retrospectively) as well as moderate levels of self-reported anxiety (30–35%, assessed with the Hospital Anxiety and Depression Scale [HADS]) or depression (13–17%, assessed with the HADS) are common neurologic and psychiatric symptoms in glioma patients [8, 9]. Multiple studies have tried to identify associations between subjective cognitive impairment, anxiety, or depression and patient-, tumour-, and treatment-related factors in glioma patients. A consistent association across studies has been found between depression on one side and poor physical functioning, reduced health-related quality of life, and decreased survival on the other [5]. However, the association between subjective cognitive impairment, anxiety, or depression and risk factors such as sex, educational level, a previous psychiatric history, and antiepileptic drugs (AEDs) in glioma patients is less clear, with existing evidence being conflicting [5, 10–14].

To prevent seizure recurrence, AEDs are generally indicated in all patients with a first seizure due to a brain tumour [7, 15]. Most AEDs are thought to have mood-modulating effects and some AEDs have been associated with the onset of depression and anxiety in epilepsy patients [16]. One of the most commonly prescribed AEDs in the glioma population, levetiracetam (LEV) [17–19], has been associated with the greatest risk of psychiatric and behavioural adverse effects compared to other AEDs [20]. Recently, three studies in the glioma population showed that LEV was associated with a higher risk of self-reported and clinician-diagnosed psychiatric adverse events, including anxiety [11, 21, 22]. Another commonly prescribed AED in the glioma population, valproic acid (VPA) [23], has been associated with decreased psychiatric and behavioural adverse effects in non-brain tumour-related epilepsy (BTRE) patients [24]. In addition, AEDs have been associated with objective as well as subjective cognitive impairment, in both epilepsy [25] and glioma patients [12, 26]. Especially the first generation of AEDs, which includes VPA, have been related to cognitive impairment in glioma patients [12, 26]. LEV, on the other hand, does not seem to have any negative effects on neurocognitive functioning of (non-)BTRE patients [27, 28]. Adverse drug effects are considerably more often reported in glioma patients compared to patients with non-BTRE [15, 19, 29]. We expect glioma patients are at higher risk of developing depression, anxiety, or cognitive adverse effects from AEDs than non-BTRE patients.

Given neuropsychiatric symptoms are frequent complications of the tumour itself and its subsequent treatment, with considerable morbidity and an adverse effect on health-related quality of life [30, 31], aim of this study was to determine whether AED use independently contributed to depression, anxiety, and subjective cognitive impairment in glioma patients. Based on previous literature, we hypothesized that: (I) use of AEDs was independently associated with self-reported depression, anxiety, and subjective cognitive impairment in glioma patients; (II) glioma patients using LEV monotherapy are more depressed and/or anxious than patients on VPA monotherapy; and (III) glioma patients using VPA monotherapy report more often subjective cognitive impairment than patients on LEV monotherapy.

## Methods

### Participants

This observational study included adult patients (≥18 years) with a histologically confirmed supratentorial grade II-IV glioma, according to the 2016 World Health Organization (WHO) classification of tumours of the central nervous system [32], who visited the neuro-oncology outpatient clinic in one of three large referral centers in the Netherlands between June 1st, 2017 and June 1st, 2018: Leiden University Medical Center in Leiden, Haaglanden Medical Center in the Hague, and the Erasmus Medical Center in Rotterdam. Patients were not eligible if they had insufficient understanding of the Dutch language in order to read the information letter and complete the self-reported questionnaires. The medical ethics committees of the participating institutions approved the study protocol and all patients provided written informed consent before participation.

### Clinical data and used instruments

Clinical data retrieved from the medical records included patient-related and tumour-related characteristics, current and previous antitumour treatment, Karnofsky Performance Status (KPS), current AED use, other prescription medications, and AED load. AED load is defined as the ratio between the prescribed daily dosage and the defined daily dosage (DDD) as defined by the WHO (Supplementary Table 1). For instance, a patient is prescribed 1500 mg VPA and 300 mg lacosamide (LCM) each day. The DDD of VPA is 1500 mg and of LCM 300 mg. His/her AED load is 2 ([1500/1500] + [300/300]) [33]. Use of prescription medications, excluding AEDs, with > 1% risk of developing depression, anxiety, or cognitive adverse effects, were extracted from the medical records. The risk of potential adverse drug reactions and treatment indications of medications was based on the Dutch ‘Farmacotherapeutisch Kompas’ (Supplementary Table 2) [34]. Patients were classified as using either none or at least one drug, separately for each of the three adverse effects. Mood stabilizing and anxiolytic medication could either be AEDs (e.g. VPA) or other prescription medications (e.g. citalopram), which were included separately as potential confounders. Medication taken as needed was excluded.

A study-specific questionnaire was used to assess other potential confounders (i.e. level of education, marital status, ethnicity, employment status, social support, history of mood disorder treatment, and mood disorder[s] in the family). Seizure severity, a potential confounding variable, was assessed with a modified version of the Liverpool Seizure Severity Scale (LSSS) [35]. Depression and anxiety symptoms were assessed with the HADS questionnaire. A cut-off of ≥8 points on the depression or anxiety domain was used to classify patients as depressed or anxious [36]. Subjective cognitive impairment was assessed with the Medical Outcomes Study-Cognitive Functioning Scale (MOS-CFS) [37]. A cut-off of ≥2 standard deviations (SD) below the mean of the reference population was used to classify patients as subjectively cognitively impaired [12, 38]. More extensive details on the questionnaires can be found in Supplementary Table 3.

### Statistical analyses

All statistical analyses were performed with SPSS 23.0 for Windows, and a p-value (*p)* < 0.05 was considered significant. A non-response analysis concerning the most important patient characteristics was performed using the χ^2^-test for proportions and the Student’s t-tests or Mann–Whitney U-test for continuous variables (depending on the distribution of the data) to assess the extent of response bias. In addition, the point prevalence rates of depression, anxiety, and subjective cognitive impairment of glioma patients was compared with normative data using the Student’s t-tests for comparison of means [37, 39].

The DAG (Directed Acyclic Graph) representation was used to identify potential confounders based on prior knowledge from the literature, meaning a confounder must be associated with both the determinant (i.e. AED use) and the outcome, but not lay in the causal path [40]. In order to assess which tumour-related, treatment-related and patient-related characteristics were associated with depression, anxiety, and subjective cognitive impairment, univariable logistic regression analyses (per outcome) with all potential confounders were performed (Supplementary Tables 4, 5, and 6). Probability for entry in the multivariable logistic regression was set at *p* < 0.10 in univariable analysis. Based on previously conducted simulation studies, a maximum of 9, 13, and 10 parameters were included in the multivariable regression model for depression, anxiety, and subjective cognitive impairment, respectively [41]. Correlation analyses were performed to identify multicollinearity, with a cut-off set at a variance inflation factor of >5.

Three multivariable logistic regression analyses were performed to identify whether use of AEDs (none versus at least one) was independently related to depression, anxiety, or subjective cognitive impairment. Previously mentioned potential confounding variables, with *p* < 0.10 in univariable logistic regression, were included. Subsequently, three additional multivariable logistic regression analyses were performed, now with a more specific definition of AED use ((1) no AED use; (2) LEV monotherapy; (3) VPA monotherapy; (4) other AED use) in order to assess if the association between AEDs and depression, anxiety, and subjective cognitive impairment differed between types of AEDs, at the expense of a loss of power. The same potential confounders as in the previous analyses were included. Two sensitivity analyses were performed with less stringent cut-offs for subjective cognitive impairment (1 SD and 1.5 SD). No sensitivity analyses were performed with the more stringent alternative cut-off (≥11 points) on the depression and anxiety domain, as this would result in an insufficient number of depressive and anxious patients to allow inclusion of confounding parameters.

## Results

Table 1 shows the sociodemographic and clinical characteristics of the included patients. A total of 536 eligible glioma patients were approached for participation, of which 272 (51%) completed the questionnaires. Most included patients were male (58%), diagnosed with glioblastoma (32%), had a partner (80%), a high level of education (43%), received radiotherapy (80%), and chemotherapy (71%). A total of 88/272 of the included patients did not use AEDs, 85 patients used LEV monotherapy, 32 patients used VPA monotherapy, 15 patients used monotherapy of other AEDs, and 52 patients used polytherapy AEDs. All 272 patients completed the questionnaires on depression, anxiety, and subjective cognition.

**Table 1:** Sociodemographic and clinical characteristics of the *n* = 272 study population

	Number of patients
Mean age in years (SD)	54 (12)
Sex, *n* (%)	
Female	113 (42%)
Male	159 (58%)
Median time since diagnosis in months (IQR)	77 (18–113)
Histological diagnosis last resection, *n* (%)	
Low-grade	135 (50%)
Diffuse astrocytoma NOS	16 (6%)
Diffuse astrocytoma IDH-mutant	36 (13%)
Oligodendroglioma NOS	7 (3%)
Oligodendroglioma IDH-mutant 1p/19q codeletion	66 (24%)
Oligoastrocytoma NOS	6 (2%)
Pleiomorphic xanthroastrocytoma	4 (1%)
High-grade	137 (50%)
Diffuse astrocytoma IDH-wildtype	5 (2%)
Anaplastic astrocytoma NOS	11 (4%)
Anaplastic astrocytoma IDH-wildtype	2 (1%)
Anaplastic astrocytoma IDH-mutant	11 (4)
Anaplastic oligodendroglioma NOS	2 (1%)
Anaplastic oligodendroglioma IDH-mutant 1p/19q codeletion	18 (7%)
Glioblastoma NOS	41 (15%)
Glioblastoma IDH-wildtype	38 (14%)
Glioblastoma IDH-mutant	9 (3%)
Extent of last resection, *n* (%)	
Biopsy	37 (14%)
Resection	227 (83%)
Missing	8 (3%)
Previously received radiotherapy, *n* (%)	
Yes	217 (80%)
No	55 (20%)
Previously received chemo- and/ or immunotherapy^a^, *n* (%)	
Temozolomide	148 (54%)
PCV	47 (21%)
Lomustine	10 (4%)
Temozolomide rechallenge	22 (8%)
Immunotherapy	8 (3%)
Other	2 (1%)
No chemo- and/or immunotherapy	79 (29%)
Tumour lobe, *n* (%)	
Frontal	162 (60%)
Non-frontal	110 (40%)
Epilepsy type, *n* (%)	
Focal	74 (27%)
Focal to bilateral tonic–clonic	48 (18%)
Focal & focal to bilateral tonic–clonic	84 (31%)
Unknown	7 (3%)
No epilepsy	59 (22%)
KPS, *n* (%)	
≥70	266 (98%)
<70	6 (2%)
Level of education, *n* (%)	
Low	72 (26%)
Medium	82 (30%)
High	118 (43%)
Ethnicity, *n* (%)	
Caucasian	252 (93%)
Other	12 (4%)
Missing	8 (3%)
Marital status, *n* (%)	
Partner	222 (82%)
No partner	50 (18%)
Current employment status, *n* (%)	
Not incapacitated to work	199 (73%)
Incapacitated to work	73 (27%)
Social support^b^, *n* (%)	
Adequate	263 (97%)
Not adequate	9 (3%)
History of mood disorder treatment (prior to glioma diagnosis), *n* (%)	
Yes^c^	31 (11%)
No	241 (89%)
Mood disorder treatment (started after glioma diagnosis), *n* (%)	
Yes^c^	33 (12%)
No	239 (88%)
Mood disorder in family^d^, *n* (%)	
Yes	79 (29%)
No	193 (71%)

*IDH* isocitrate dehydrogenase, *IQR* interquartile range, *KPS* Karnofsky performance status, *NOS* not otherwise specified, *SD* standard deviation

^a^Percentages do not add-up to 100%, since patients could have received more than one type of chemo- and/or immunotherapy

^b^Social support was measured with two questions (yes/no) concerning if patient had friends or family that can help when you need them and you can speak to confidentially (not adequate social support =  ≥ 1 no);

^c^Psychologically and/ or medically

^d^First and/ or second degree relatives with diagnosis of depression, anxiety or bipolar disorder

The non-response analysis showed that patients who participated had less often KPS scores < 70 (2% versus 9%, *p* = 0.001) and a higher mean age (54 [SD = 13] versus 50 [SD = 12] years, *p* = 0.001) compared to patients who did not participate in the study, while they did not differ significantly on other patient- and disease-related characteristics.

### Depression

Glioma patients had a significantly higher mean depression score when compared with Dutch normative data (4.1 [SD = 3.9] versus 3.4 [SD = 3.3], respectively, *p* = 0.006), but this difference was not considered clinically relevant [39]. A total of 47/272 (17%) patients were considered depressed. Prevalence of depression differed significantly between patients not using (10%) and using AEDs (21%, unadjusted Odds Ratio [uOR] = 2.29 [95%CI 1.05–4.97], *p* = 0.037), but this significant difference disappeared after adjustment for potential confounders (adjusted Odds Ratio [aOR] = 1.94 [95%CI 0.83–4.50], *p* = 0.125). Use of prescription medications with > 1% risk of depressive adverse effects (excluding AEDs) was still independently associated with a higher prevalence of depression after adjustment for confounders, which was true as well for being incapacitated to work and KPS score < 70 (Table 2).

**Table 2:** Unadjusted and adjusted odds ratios of the predictor variables of depression in the multivariable analysis

Parameter^a^		Depression (≥ 8 points on the HADS-D)					
		uOR	95% CI	*p*-value	aOR	95% CI	*p*-value
Current AED use, dichotomised	No AEDs (ref.)						
	≥1	2.29	1.05–4.97	0.037*	1.94	0.83–4.50	0.125
Medications > 1% risk of DAEs^b^	None (ref.)						
	≥1	2.18	1.14–4.19	0.019*	2.27	1.12–4.62	0.024*
Seizure severity		1.03	1.00–1.07	0.055	1.02	0.99–1.06	0.251
Level of education	Low (ref.)						
	Medium/ high	2.84	1.15–7.00	0.024*	2.18	0.85–5.59	0.105
Employment status	Not incapacitated to work (ref.)						
	Incapacitated to work	2.15	1.11–4.15	0.023*	2.01	0.99–4.06	0.052
Most recent tumour grade^c^	Low (grade II, ref.)						
	High (grade III & IV)	0.50	0.26–0.95	0.034*	0.50	0.25–1.03	0.059
KPS	≥ 0 (ref.)						
	<70	10.37	1.84–58.42	0.008*	9.34	1.53–56.90	0.015*

*AED* Antiepileptic drug, *aOR* adjusted odds ratio, *CI* confidence interval, *DAEs* depressive adverse effects, *HADS-D* hospital anxiety and depression scale-depression subscale, *KPS* Karnofsky performance status, *ref.* reference category, *uOR* unadjusted odds ratio

*p < 0.05;

^a^Univariable analyses on all predictor variables of depression in this study can be found in the supplementary Table 4

^b^Excluding AEDs

^c^Diffuse astrocytoma isocitrate dehydrogenase (IDH)-wildtype was considered high-grade

We hypothesized that patients using LEV monotherapy were more depressed than patients using VPA monotherapy. However, the prevalence of depression was not significantly higher for LEV monotherapy (22%) compared to VPA monotherapy (19%), neither before or after adjustment for potential confounders (aOR = 0.76 [95%CI 0.26–2.23, *p* = 0.616). No significant differences were found comparing LEV monotherapy with patients not using AEDs (10%) or other AEDs (19%), neither before or after correction for potential confounders [Supplementary Table 7]).

### Anxiety

The mean anxiety score of glioma patients was not significantly different from Dutch normative data (5.0 [SD = 3.7] versus 5.1 [SD = 3.6], *p* = 0.535) [39]. A total of 64 (24%) of all 272 included glioma patients were considered anxious. Prevalence of anxiety did not differ significantly between patients not using (19%) and using AEDs (26%, uOR = 1.43 [95%CI 0.77–2.68], *p* = 0.259) and adjustment for confounders did not alter the results (aOR = 1.17 [95%CI 0.59–2.29], *p* = 0.659 [Table 3]). Only history of mood disorder treatment was independently associated with anxiety after correction of confounders.

**Table 3:** Unadjusted and adjusted odds ratios of the predictor variables of anxiety in the multivariable analysis

Parameter^a^		Anxiety (≥ 8 points on the HADS-A)					
		uOR	95% CI	*p*-value	aOR	95% CI	*p*-value
Current AED use, dichotomised	No AEDs (ref.)						
	≥1	1.43	0.77–2.68	0.259	1.17	0.59–2.29	0.659
Seizure severity		1.03	1.00–1.07	0.044*	1.03	1.00–1.06	0.091
Age		0.98	0.96–1.00	0.075	0.98	0.96–1.01	0.194
Ethnicity	Caucasian (ref.)						
	Other	3.50	1.09–11.28	0.036*	3.17	0.94–10.75	0.064
Social support	Adequate (ref.)						
	Not adequate	4.32	1.13–16.61	0.033*	3.73	0.86–16.26	0.080
History of mood disorder treatment^b^	No (ref.)						
	Yes	3.15	1.45–6.81	0.004*	2.76	1.23–6.19	0.014*

*AED* Antiepileptic drug, *aOR* adjusted odds ratio, *CI* confidence interval, *HADS-A* hospital anxiety and depression scale-anxiety subscale, *ref.* reference category, *uOR* unadjusted odds ratio

*p < 0.05;

^a^Univariable analyses on all predictor variables of anxiety in this study can be found in the supplementary Table 5

^b^Prior to glioma diagnosis

We hypothesized that patients using LEV monotherapy were more anxious than patients using VPA monotherapy. When comparing LEV with other AEDs or patients not using AEDs, prevalence of anxiety was not significantly higher for LEV monotherapy (32%), VPA monotherapy (16%, aOR = 0.55 [95%CI 0.19–1.65], *p* = 0.289), other AEDs (22%), or patients not using AEDs (19%), neither before nor after adjustment of potential confounders (Supplementary Table 8).

### Subjective cognitive functioning

The mean subjective cognitive functioning score of glioma patients was significantly lower than normative data (66.9 [SD = 21.3] versus 81.9 [SD = 16.9], *t*(271) = −11.64, *p* < 0.001) [37]. A total of 19% (52/272) of patients were considered subjectively cognitively impaired. Prevalence of subjective cognitive impairment did not differ between patients not using (16%) and using AEDs (21%, uOR = 1.38 [95%CI 0.70–2.70], *p* = 0.353) and adjustment of confounders did not alter the results (aOR = 0.83 [95%CI 0.34–2.04], *p* = 0.684 [Table 4]). Solely seizure severity was independently associated with subjective cognitive impairment after correction of confounders. Alternate cut-offs for subjective cognitive dysfunction did not result in different results (data not shown).

**Table 4:** Unadjusted and adjusted odds ratios of the predictor variables of subjective cognitive impairment in the multivariable analysis

Parameter^a^		Impaired subjective cognition (≥ 2SD below the mean of normative data from the MOS)					
		uOR	95% CI	*p*-value	aOR	95% CI	*p*-value
Current AED use, dichotomised	No AEDs (ref.)						
	≥1	1.38	0.70–2.70	0.353	0.83	0.34–2.04	0.684
Medications > 1% risk of CAEs^b^	None (ref.)						
	≥1	2.34	1.09–5.04	0.030*	2.18	0.97–4.88	0.059
Seizure severity		1.04	1.01–1.08	0.012*	1.04	1.00–1.07	0.044*
Total AED load		1.36	0.98–1.91	0.070	1.31	0.84–2.05	0.236
Sex	Female (ref.)						
	Male	0.59	0.32–1.09	0.093	0.61	0.32–1.15	0.125
Social support	Adequate (ref.)						
	Not adequate	3.58	0.93–13.84	0.064	2.38	0.53–10.80	0.260
Mood disorder in family^c^	No (ref.)						
	Yes	1.89	1.01–3.55	0.047*	1.53	0.77–3.00	0.223

*AED* Antiepileptic drug, *aOR* adjusted odds ratio, *CAEs* cognitive adverse effects, *CI* confidence interval, *MOS* medical outcomes study, *ref.* reference category, *uOR* unadjusted odds ratio

*p < 0.05;

^a^Univariable analyses on all predictor variables of subjective cognitive impairment in this study can be found in the supplementary table 6

^b^Excluding AEDs

^c^First and/ or second degree relatives

We hypothesized that patients using VPA monotherapy reported more often subjective cognitive impairments than patients using LEV monotherapy. The prevalence of subjective cognitive impairment was not significantly higher for VPA monotherapy (28%) compared to LEV monotherapy (14%, aOR = 0.40 [95%CI 0.14–1.11], *p* = 0.078), other AEDs (25%), or patients not using AEDs (16%), neither before nor after adjustment for potential confounders (Supplementary Table 9). Alternate cut-offs did not give different results (data not shown).

## Discussion

Previous studies have shown that depression (13–17%), anxiety (30–35%), and subjective cognitive impairment (80%) frequently occur in glioma patients [8, 9]. Numerous factors can be the causative or contributing factor of these impactful symptoms in glioma patients [30, 31, 42], including AEDs [12, 16, 20, 25, 26]. The above mentioned neuropsychiatric symptoms are commonly reported as adverse effects of AEDs and glioma patients seem to be more vulnerable for adverse drug reactions of AEDs compared to patients with non-BTRE [15, 19, 29]. Therefore, we hypothesized that AED use is independently associated with self-reported depression, anxiety, and subjective cognitive impairment in glioma patients. In addition, we hypothesized patients on LEV would have an increased risk for depression and anxiety, while patients on VPA would have an increased risk for subjective cognitive impairment. The findings in this study, however, do not support any of the three hypotheses. Although we found that the prevalence of depression was significantly higher in patients using AEDs compared to patients not using AEDs, this effect disappeared after adjustment for potential confounders, suggesting that the risk of depression is caused by other factors than AED use. Thereby, a lack of sufficient statistical power might have played a role in the absence of a statistically significant difference between AED types.

LEV has generally become one of the preferred AEDs in glioma patients due to the lack of any known pharmacological interactions [15]. A perceived higher risk of psychiatric adverse effects in patients on LEV is a concern of physicians and sometimes a reason to choose another AED over LEV [11, 43]. Similar considerations apply to VPA with regard to a perceived higher risk of cognitive adverse effects [12]. Our data showed that the risk of having depression, anxiety, or subjective cognitive impairment does not significantly differ between patients on LEV, VPA, other AEDs and patients not using AEDs. Therefore, choosing certain AEDs over others or withholding AEDs in order to reduce the risk of depression, anxiety, or subjective cognitive impairment does in general not seem to be justified by our results. Nevertheless, on an individual basis different choices can be made.

Our results are in contrast with other studies in brain tumour patients, demonstrating that LEV had an increased risk for psychiatric adverse effects, including anxiety [11, 21, 22]. This might be partly due to differences in patient populations [22], the instrument used for measurement of anxiety [11, 21], and/ or adjustment of different confounding variables [11, 21, 22]. Nonetheless it remains unclear why certain confounding variables in other studies, such as a tumour in the frontal lobe [11, 21, 22], were not related to depression and/or anxiety in our study. We found that both prescription medications (excluding AEDs) with >1% risk of depression as adverse effect and poor performance status were the most important contributing factors for developing depression. In case of anxiety, a history of mood disorder treatment was the most contributing factor, and in case of subjective cognitive impairment it was seizure severity. Particularly the use of prescription medications other than AEDs with a risk of developing depression is of interest, as this could easily be adapted by a physician. Replacing medication with a relevant risk of depression as adverse effect by medication with a lower risk, should be considered at a low threshold in glioma patients with depressive mood symptoms. For instance, a dopamine-antagonist such as metoclopramide, which has a >1% risk of depression as adverse effect, can be exchanged for a 5HT3-antagonist like ondansetron as anti-emetic prophylaxis for chemotherapy induced nausea and vomiting. The limited use of older AEDs, such as phenytoin and phenobarbital, which are known for their cognitive adverse effects [26], might explain the absence of an association in our study between AED use and subjective cognitive impairment, which is in contrast to what has been reported previously [12]. Of note, LEV has even been associated with an improved verbal memory in glioma patients [28], although cognitive functioning was measured objectively instead of subjectively as in our study. Typically, the correlation between subjective and objective measures of cognition is regarded low, with subjective cognitive symptoms being more closely related to emotional and mental symptoms [10].

Nevertheless, our findings need to be interpreted carefully. In at least 13 patients treatment with LEV, VPA, and/ or topiramate was discontinued or adjusted due to psychiatric adverse effects related to the AED, according to the treating physician. Moreover, only 32 patients used VPA monotherapy and a lack of statistical power might have played a role in the absence of an association between VPA and subjective cognitive impairment. The prevalence of subjective cognitive impairment was twice as high in patients using VPA monotherapy (28%) compared to LEV monotherapy (14%) or no AEDs (16%). Due to the cross-sectional nature of our observational study we cannot establish or refute a definitive causal link between AED use and concurrent depression, anxiety, or subjective cognitive impairment in glioma patients. Despite including a wide variety of potential confounders in this study, residual confounding might still be present as some potential confounders were not incorporated in the analysis included (e.g. pre-existing conditions with a comorbidity index). An ongoing randomized controlled clinical trial also assessing depression, anxiety, and subjective cognitive impairment in patients on LEV versus VPA may contribute to elucidate this issue (ClinicalTrials.gov Identifier: NCT03048084).

A strength of our study is that we included all types of diffuse glioma patients and did not exclude certain patients, such as patients with a (family) history of psychiatric disorder [11], but instead included this as a potential confounder. In addition, we included prescription medications other than AEDs with >1% risk of depression as a relevant confounder, which has not been reported before, and found an association with a higher risk of depression. Although the non-response analysis showed that the percentage of patients in the study population with poor performance status was significantly lower and the mean age higher, the actual differences were not clinically relevant. Therefore, our results can be considered generalizable to the general glioma population.

## Conclusion

Our results suggest that AED use was not associated with a higher risk of developing depression, anxiety, or subjective cognitive impairment in glioma patients, as there were no significant differences between patients using and not using AEDs, or between different types of AEDs. The risk of having depression, anxiety, or subjective cognitive impairment in glioma patients seems mainly be related to alternative factors. Based on these findings, choosing certain AEDs over others solely in order to reduce the risk of depression, anxiety, or subjective cognitive impairment does not seem to be justified. However, results from larger, preferably prospective, studies are needed to confirm our findings.

## Supplementary Information

Below is the link to the electronic supplementary material.

Supplementary file1 (DOCX 81 kb)

## Data Availability

Data are available upon reasonable request.
