# Supplementary material for: Effect of antiepileptic drugs in glioma patients on self-reported depression, anxiety, and cognitive complaints
Source: J Neurooncol. 2021 Apr 6;153(1):89–98. doi: 10.1007/s11060-021-03747-1 (PMC8131297; doi:10.1007/s11060-021-03747-1)
Supplement: Supplementary file 1 — Supplementary file1 (DOCX 81 kb) [file 11060_2021_3747_MOESM1_ESM.docx]

**Supplementary Material**

**Effect of Antiepileptic Drugs in Glioma Patients on Self-Reported Depression, Anxiety, and Cognitive Complaints**

Pim B. van der Meer, Johan A.F. Koekkoek, Martin J. van den Bent, Linda Dirven, Martin J.B. Taphoorn

**Content:**

**Supplementary Table 1.** List with defined daily dosages, as defined by the World Health Organisation, of antiepileptic drugs prescribed in this study.

**Supplementary Table 2.** Medications prescribed to glioma patients in our study with corresponding

depressive, anxiety and cognitive adverse effects.

**Supplementary Table 3.** Detailed information on the used questionnaires.

**Supplementary Table 4.** Univariable analyses of predictor variables of depression.

**Supplementary Table 5.** Univariable analyses of predictor variables of anxiety.

**Supplementary Table 6.** Univariable analyses of possible confounding predictor variables of subjective cognitive impairment.

**Supplementary Table 7.** Unadjusted and adjusted odds ratios of the predictor variables of depression with current AED use specified in the multivariable analysis.

**Supplementary Table 8.** Unadjusted and adjusted odds ratios of the predictor variables of anxiety with current AED use specified in the multivariable analysis.

**Supplementary Table 9.** Unadjusted and adjusted odds ratios of the predictor variables of subjective cognitive impairment with current AED use specified in the multivariable analysis.

**References.**

**Supplementary Table 1.** List with defined daily dosages, as defined by the World Health Organisation, of antiepileptic drugs prescribed in this study

Antiepileptic drug Defined daily dosage Unit

Carbamazepine 1 g

Clonazepam 8 mg

Clobazam 20 mg

Gabapentin 1.8 g

Lacosamide 0.3 g

Lamotrigine 0.3 g

Levetiracetam 1.5 g

Phenytoin 0.3 g

Pregabalin 0.3 g

Topiramate 0.3 g

Valproic acid 1.5 g

Zonisamide 0.2 g

G=gram, mg=milligram

**Supplementary Table 2.** Medications prescribed to glioma patients in our study with corresponding

depressive, anxiety and cognitive adverse effects

|  |  | **Adverse effects** | | |
| --- | --- | --- | --- | --- |
| **Medication** | **No. of patients (n=272)** | **Depressive^1^** | **Anxiety^2^** | **Cognitive^3^** |
| Acenocoumarol | 7 | - | - | - |
| Acetylsalicyl acid | 1 | - | Reported | Reported |
| Alendronic acid | 4 | - | - | - |
| Alendronic acid/colecalciferol | 2 | - | - | - |
| Alfacalcidol | 2 | - | - | 0,1-1% |
| Alfuzosin | 1 | - | - | - |
| Aliskiren | 1 | - | - | - |
| Allopurinol | 1 | <0,01% | - | - |
| Amantadine | 1 | **>1%** | **>1%** | **>1%** |
| Amitryptiline* | 3 | 0,01-0,1% | 0,1-1% | **>1%** |
| Amlodipine | 11 | 0,1-1% | - | 0,01-0,1% |
| Apixaban | 1 | - | - | - |
| Aripiprazole | 1 | 0,1-1% | **>1%** | - |
| Atorvastatin | 6 | Reported | - | - |
| Azelastine/fluticasone | 1 | - | - | - |
| Barnidipine | 2 | - | - | - |
| Beclomethasone | 2 | Reported | Reported | - |
| Beclomethasone/formoterol | 2 | Reported | 0,1-1% | - |
| Biperiden | 1 | - | 0,01-0,1% | 0,01-0,1% |
| Bisoprolol | 1 | 0,1-1% | - | - |
| Budesonide | 1 | 0,1-1% | 0,1-1% | - |
| Cabergoline | 1 | **>1%** | - | - |
| Calcipotriol/betamethasone | 1 | - | - | - |
| Calcium carbonate | 2 | - | - | - |
| Calcium carbonate/colecalciferol | 8 | - | - | - |
| Carbamazepine*^/4^ | 13 | 0,1-1% | - | <0,01% |
| Carbasalate calcium | 9 | - | - | - |
| Carbomer | 1 | - | - | - |
| Celecoxib | 4 | 0,1-1% | 0,1-1% | 0,01-0,1% |
| Cetirizine | 1 | 0,01-0,1% | - | 0,01-0,1% |
| Cholecalciferol or colecalciferol | 9 | - | - | - |
| Ciclesonide | 1 | Reported | Reported | - |
| Citalopram*^/^** | 1 | - | **>1%** | **>1%** |
| Clemastine | 1 | - | - | - |
| Clobazam** | 5 | **>1%** | 0,1-1% | **>1%** |
| Clonazepam | 6 | **>1%** | **>1%** | **>1%** |
| Clopidogrel | 9 | - | - | <0,01% |
| Codeine | 1 | Reported | Reported | reported |
| Co-trimoxazol | 7 | Reported | - | - |
| Cromoglicic acid | 1 | - | - | - |
| Dabigatran | 1 | - | - | - |
| Dalteparin | 1 | - | - | - |
| Desloratidine | 2 | - | - | - |
| Dexamethasone | 33 | **>1%** | **>1%** | - |
| Dextran/hypromellose | 1 | - | - | - |
| Diazepam** | 2 | **>1%^6^** | - | **>1%** |
| Diclofenac | 4 | <0,01% | <0,01% | <0,01% |
| Diltiazem | 2 | - | 0,1-1% | - |
| Doxazosin | 1 | 0,1-1% | 0,1-1% | - |
| Dutasteride | 1 | Reported | - | - |
| Enalapril | 4 | **>1%** | 0,1-1% | 0,1-1% |
| Esomeprazole | 4 | 0,01-0,1% | - | 0,01-0,1% |
| Estradiol | 1 | **>1%** | 0,1-1% | - |
| Estradiol/dydrogesterone | 2 | **>1%** | **>1%^7^** | - |
| Etanercept | 1 | Reported | - | - |
| Ethinylestradiol/levonorgestrel | 2 | **>1%** | **>1%^7^** | - |
| Etoricoxib | 4 | 0,1-1% | 0,1-1% | 0,1-1% |
| Ezetimibe | 2 | - | - | - |
| Ezetimibe/simvastatin | 1 | Reported | - | Reported |
| Ezetimibe/atorvastatin | 1 | 0,1-1% | - | Reported |
| Finasteride | 2 | 0,1-1% | - | - |
| Flecainide | 1 | 0,01-0,1% | 0,1-1% | 0,1-1% |
| Fluorouracil | 1 | - | - | - |
| Fluoxetine*^/^** | 1 | 0,1-1% | **>1%** | **>1%** |
| Fluticasone | 4 | <0,01% | <0,01% | - |
| Folic acid | 2 | <0,01% | - | - |
| Formoterol | 1 | - | 0,1-1% | - |
| Fosinopril | 1 | **>1%^6^** | - | Reported |
| Furosemide | 1 | - | - | - |
| Fusidic acid | 1 | - | - | - |
| Gabapentin | 4 | **>1%** | **>1%** | **>1%** |
| Gemfibrozil | 1 | 0,01-0,1% | - | - |
| Gliclazide | 2 | - | - | - |
| Glycopyrronnium bromide | 1 | **>1%^6^** | Reported | **>1%** |
| Granisetron | 5 | - | - | - |
| Hydrochlorothiazide | 11 | 0,01-0,1% | - | - |
| Hydroxychloroquine | 1 | - | 0,01-0,1% | - |
| Hypromellose | 1 | - | - | - |
| Indapamide | 1 | - | - | - |
| Insulin aspart | 2 | - | - | - |
| Insulin glargine | 1 | - | - | - |
| Ipratropium bromide | 1 | - | - | - |
| Irbesartan | 3 | - | - | - |
| Isosorbide dinitrate | 2 | - | - | - |
| Itraconazole | 1 | - | - | **>1%** |
| Ketoconazole | 1 | - | - | - |
| Lacosamide | 15 | **>1%** | 0,1-1% | **>1%** |
| Lactulose | 5 | - | - | - |
| Lamotrigine* | 12 | - | - | <0,01% |
| Letrozole | 1 | **>1%** | 0,1-1% | 0,1-1% |
| Levetiracetam | 122 | **>1%** | **>1%** | 0,1-1% |
| Levocetirizine | 1 | Reported | Reported | - |
| Levodopa/carbidopa | 3 | **>1%** | - | **>1%** |
| Levonorgestrel | 1 | **>1%** | **>1%^7^** | - |
| Levothyroxine | 7 | - | - | - |
| Lidocaine | 1 | - | - | - |
| Lisinopril | 4 | 0,1-1% | - | 0,01-0,1% |
| Loratidine | 1 | - | **>1%^7^** | - |
| Lorazepam** | 1 | 0,1-1% | - | 0,1-1% |
| Losartan | 5 | Reported | - | - |
| Losartan/hydrochloorthiazide | 1 | 0,1-1% | 0,1-1% | - |
| Macrogol | 19 | - | - | - |
| Magnesium hydroxide | 1 | - | - | - |
| Melatonin | 1 | 0,01-0,1% | 0,1-1% | 0,01-01% |
| Mesalazine | 1 | Reported | - | - |
| Metformin | 10 | - | - | - |
| Methotrexate | 2 | 0,1-1% | - | - |
| Methylphenidate | 4 | **>1%** | **>1%** | - |
| Metoclopramide | 8 | **>1%** | - | 0,01-0,1% |
| Metoprolol | 11 | 0,1-1% | 0,01-0,1% | 0,1-1% |
| Metronidazole | 1 | - | - | - |
| Miconazole | 1 | - | - | - |
| Midazolam | 2 | **>1%^6^** | - | **>1%** |
| Minocycline | 1 | - | - | Reported |
| Mirtazapine* | 2 | Reported | **>1%** | **>1%** |
| Mometasone furoate | 2 | - | - | - |
| Montelukast | 2 | 0,1-1% | 0,1-1% | 0,01-0,1% |
| Nadroparin calcium | 4 | - | - | - |
| Naproxen | 1 | 0,01-0,1% | - | 0,01-0,1% |
| Nifedipine | 1 | <0,01% | 0,1-1% | - |
| Nitrofurantoin | 1 | Reported | - | - |
| Norethisterone | 1 | <0,01% | - | - |
| Octreotide | 1 | - | - | - |
| Olanzapine | 2 | **>1%^6^** | - | - |
| Omeprazole | 21 | 0,01-0,1% | 0,01-0,1% | - |
| Ondansetron | 1 | - | - | - |
| Oxazepam** | 6 | **>1%^6^** | - | **>1%** |
| Oxycodone | 3 | **>1%** | **>1%** | **>1%** |
| Pantoprazole | 28 | 0,01-0,1% | - | <0,01% |
| Paracetamol^5^ | - | 0,1-1% | - | 0,1-1% |
| Paroxetine*^/^** | 2 | Reported | **>1%^7^** | **>1%** |
| Pegvisomant | 1 | - | 0,1-1% | 0,1-1% |
| Perindopril | 7 | 0,1-1% | - | <0,01% |
| Phenytoin | 3 | - | **>1%^7^** | **>1%** |
| Polystyrene sulfonate | 1 | - | - | - |
| Pramipexole | 2 | Reported | Reported | **>1%** |
| Pravastatin | 5 | Reported | - | Reported |
| Prednisolone | 4 | **>1%** | **>1%** | - |
| Pregabalin** | 4 | 0,1-1% | 0,1-1% | **>1%** |
| Procarbazine | 1 | **>1%** | - | **>1%** |
| Propranolol** | 3 | 0,01-0,1% | - | 0,01-0,1% |
| Pyridoxine | 1 | - | - | - |
| Ranitidine | 1 | <0,01% | - | <0,01% |
| Ropinirole | 1 | Reported | **>1%^7^** | **>1%** |
| Salbutamol | 5 | - | - | - |
| Salmeterol/fluticasone | 1 | - | 0,1-1% | - |
| Sertraline*^/^** | 1 | **>1%** | **>1%** | **>1%** |
| Sevelamer | 1 | - | - | - |
| Sildenafil | 1 | - | - | - |
| Simvastatin | 12 | Reported | - | <0,01% |
| Solifenacin | 1 | - | - | - |
| Sotalol | 4 | **>1%** | **>1%** | - |
| Spironolactone | 1 | - | - | 0,1-1% |
| Sulfasalazine | 1 | 0,1-1% | <0,01% | - |
| Tacrolimus | 1 | **>1%** | **>1%** | **>1%** |
| Tamsulosin | 6 | - | - | - |
| Temazepam | 3 | **>1%^6^** | - | **>1%** |
| Testosterone | 2 | **>1%^6^** | Reported | - |
| Ticagrelor | 1 | - | - | 0,1-1% |
| Timolol/bimatoprost | 1 | 0,1-1% | - | 0,01-0,1% |
| Tiotropium bromide | 1 | - | - | - |
| Topiramate | 10 | **>1%** | **>1%** | **>1%** |
| Tramadol | 6 | 0,01-0,1% | 0,01-0,1% | 0,01-0,1% |
| Trazodone* | 1 | Reported | **>1%^7^** | **>1%** |
| Triamcinolone | 1 | - | - | - |
| Ursodeoxycholic acid | 1 | - | - | - |
| Valproic acid* | 53 | Reported | - | **>1%** |
| Valsartan | 3 | - | - | - |
| Venlafaxine*^/^** | 5 | 0,1-1% | - | **>1%** |
| Vildagliptin/metformin | 1 | - | - | - |
| Zonisamide | 1 | **>1%** | **>1%** | **>1%** |

^1^The following adverse effects were considered depressive: depression, flat affect, lethargy, apathy, suicidal thoughts, mood disorder and mood swings; ^2^The following adverse effects were considered anxiety: anxiety, nervousness, agitation and panic (attacks); ^3^The following adverse effects were considered cognitive: cognitive impairment, concentration disorder, confusion, amnesia, attention disorder, reduced memory, bradyphrenia, aphasia, reduced alertness and disorientation; ^4^Carbamazepine was considered as a medication with >1% risk of cognitive adverse effects, despite the percentages on Farmacotherapeutisch Kompas, based on literature;(1, 2) ^5^We did not include paracetamol in our analyses, as we were not confident this was reported adequately in the medical record; ^6^Closely related adverse effect of depression and considered depressive, only indicated if >1%; ^7^Closely related adverse effect of anxiety and considered as an anxiety adverse effect, only indicated if >1%;

*These medications have a mood stabilizing indication according to Farmacotherapeutisch Kompas; **These medications have an anxiolytic indication according to Farmacotherapeutisch Kompas,

**Supplementary Table 3.** Detailed information on the used questionnaires

| **Questionnaire** | **Explanation** |
| --- | --- |
| Study specific questionnaire | The following potential confounders were assessed in the study specific questionnaire: ethnicity, level of education, marital status, current employment status, availability of social support, prior depressive or anxiety disorder, and family history of mood disorders |
| Liverpool Seizure Severity Scale (LSSS) | Seizure severity was measured with a modified version of the Liverpool Seizure Severity Scale (LSSS), evaluating the seizure severity during the past four weeks. The questionnaire contains 12 items about seizure severity. The total severity score, after a linear transformation of the sum of responses, is expressed in a score ranging from 0 (no seizures) to 100 (most severe possible).(3) |
| Hospital Anxiety and Depression Scale (HADS) | This 14-item self-assessment scale consists of seven items related to depression and seven items related to anxiety. A cut-off of ≥8 points (range 0-21) on the depression or anxiety domain was used to classify patients dichotomously as depressed or anxious, as this is seen as the preferred cut-off for detecting clinical depression and anxiety in the setting of an outpatient clinic.(4, 5) Missing items were imputed by the subject’s mean if at least half of items were answered.(6) |
| Medical Outcomes Study-Cognitive Functioning Scale (MOS-CFS) | The CFS includes six questions of less severe, day-to-day problems including reasoning, concentration and thinking, memory, attention and psychomotor function. The raw scores of this self-reported CFS were converted linearly to a 0-100 scale, with higher scores indicating less cognitive complaints.(7) Subsequently, these individual scores were converted into z-scores, based on the normative scores from the MOS study and matched on age. Subjective cognitive impairment was defined as 2 standard deviations (SD) below the mean of the reference population. Similar to previous studies in glioma patients, subjective cognitive impairment was defined as a categorical variable: 2 standard deviations (SD) below the mean of the reference population.(1, 8) |

**Supplementary Table 4.** Univariable analyses of predictor variables of depression

|  |  | **Depression (≥8 points on the HADS-D)** | | | |
| --- | --- | --- | --- | --- | --- |
| Parameter |  | No./total (%) | uOR | 95% CI | p-value |
| Current AED use, dichotomised | No AEDs (ref.) | 9/88 (10%) |  |  |  |
|  | ≥1 | 38/184 (21%) | 2.29 | 1.05-4.97 | 0.037* |
| Current AED use, specified | Monotherapy LEV | 19/85 (22%) |  |  |  |
|  | Monotherapy VPA | 6/32 (19%) | 0.80 | 0.29-2.23 | 0.672 |
|  | Other | 13/67 (19%) | 0.84 | 0.38-1.85 | 0.658 |
|  | No AEDs | 9/88 (10%) | 0.40 | 0.17-0.93 | 0.034* |
| Medications >1% risk of DAEs, excluding AEDs | No (ref.) | 27/195 (14%) |  |  |  |
|  | Yes | 20/77 (26%) | 2.18 | 1.14-4.19 | 0.019* |
| Mood stabilizing medication^1^ | No (ref.) | 35/197 (18%) |  |  |  |
|  | Yes | 12/75 (16%) | 0.88 | 0.43-1.81 | 0.731 |
| Total AED load |  | 47/271 (17%) | 1.26 | 0.89-1.79 | 0.195 |
| Seizure severity^2^ |  | 47/272 (17%) | 1.03 | 1.00-1.07 | 0.055* |
| Status epilepticus^3^ | No (ref.) | 35/176 (20%) |  |  |  |
|  | Yes | 6/33 (18%) | 0.90 | 0.34-2.34 | 0.821 |
| Age |  | 47/272 (17%) | 1.00 | 0.97-1.03 | 0.948 |
| Sex | Female (ref.) | 18/113 (16%) |  |  |  |
|  | Male | 29/159 (18%) | 1.18 | 0.62-2.24 | 0.620 |
| Time since diagnosis |  | 47/272 (17%) | 1.00 | 0.99-1.00 | 0.360 |
| Ethnicity | Caucasian (ref.) | 42/252 (17%) |  |  |  |
|  | Other | 3/12 (25%) | 1.67 | 0.43-6.42 | 0.458 |
| Level of education | Low (ref.) | 6/72 (8%) |  |  |  |
|  | Medium/ high | 41/200 (21%) | 2.84 | 1.15-7.00 | 0.024* |
| Marital status | Partner (ref.) | 38/222 (17%) |  |  |  |
|  | No partner | 9/50 (18%) | 1.06 | 0.48-2.37 | 0.881 |
| Employment status | Not incapacitated to work (ref.) | 28/199 (14%) |  |  |  |
|  | Incapacitated to work | 19/73 (26%) | 2.15 | 1.11-4.15 | 0.023* |
| Social support | Adequate (ref.) | 44/263 (17%) |  |  |  |
|  | Not adequate | 3/9 (33%) | 2.49 | 0.60-10.33 | 0.209 |
| History of mood disorder treatment^4^ | No (ref.) | 39/241 (16%) |  |  |  |
|  | Yes | 8/31 (26%) | 1.80 | 0.75-4.32 | 0.187 |
| Mood disorder in family^5^ | No (ref.) | 35/193 (18%) |  |  |  |
|  | Yes | 12/79 (15%) | 0.81 | 0.40-1.65 | 0.560 |
| Most recent tumour grade^6^ | Low (grade II, ref.) | 30/135 (22%) |  |  |  |
|  | High (grade III & IV) | 17/137 (12%) | 0.50 | 0.26-0.95 | 0.034* |
| Extent of last resection | Biopsy (ref.) | 3/37 (8%) |  |  |  |
|  | Resection | 43/228 (19%) | 2.63 | 0.77-8.98 | 0.122 |
| Radiotherapy | No (ref.) | 12/55 (22%) |  |  |  |
|  | Yes | 35/217 (16%) | 0.69 | 0.33-1.44 | 0.321 |
| Chemo- and or immunotherapy | No (ref.) | 14/79 (18%) |  |  |  |
|  | Yes | 33/193 (17%) | 0.96 | 0.48-1.91 | 0.902 |
| Tumour lobe | Non-frontal (ref.) | 17/110 (15%) |  |  |  |
|  | Frontal | 30/162 (19%) | 1.24 | 0.65-2.39 | 0.512 |
| KPS | ≥70 (ref.) | 43/266 (16%) |  |  |  |
|  | <70 | 4/6 (67%) | 10.37 | 1.84-58.42 | 0.008* |

^1^Excluding mood stabilizing medication for treatment of depression first prescribed after glioma diagnosis; ^2^Score 0-100 measured with the Liverpool Seizure Severity Scale; ^3^Status epilepticus was defined as ongoing seizures for ≥30 minutes if convulsive or ≥60 minutes if non-convulsive, because it has been thought long-term consequences might occur after this timeframe; ^4^Prior to glioma diagnosis, treatment started after glioma diagnosis was considered potentially in the causal pathway, as most medications with depressive and anxiety adverse effects were started after glioma diagnosis, and therefore not further analysed; ^5^First and/ or second degree relatives; ^6^Diffuse astrocytoma isocitrate dehydrogenase (IDH)-wildtype was considered high-grade; **p*<0.1; AEDs=Antiepileptic drugs; CI=Confidence Interval; DAEs=Depressive Adverse Effects; HADS-D=Hospital Anxiety and Depression Scale-Depression subscale; KPS=Karnofsky Performance Status; LEV=Levetiracetam; ref.=reference category; uOR=unadjusted Odds Ratio; VPA=Valproic acid

**Supplementary Table 5.** Univariable analyses of predictor variables of anxiety

|  |  | **Anxiety (≥8 points on the HADS-A)** | | | |
| --- | --- | --- | --- | --- | --- |
| Parameter |  | No. (%) | uOR | 95% CI | p-value |
| Current AED use, dichotomised | No AEDs (ref.) | 17/88 (19%) |  |  |  |
|  | ≥1 | 47/184 (26%) | 1.43 | 0.77-2.68 | 0.259 |
| Current AED use, specified | Monotherapy LEV (ref.) | 27/85 (32%) |  |  |  |
|  | Monotherapy VPA | 5/32 (16%) | 0.40 | 0.14-1.15 | 0.088* |
|  | Other | 15/67 (22%) | 0.62 | 0.30-1.29 | 0.201 |
|  | No AEDs | 17/88 (19%) | 0.51 | 0.26-1.04 | 0.062* |
| Medications >1% risk of AAEs, excluding AEDs | No (ref.) | 48/213 (23%) |  |  |  |
|  | Yes | 16/59 (27%) | 1.28 | 0.66-2.47 | 0.463 |
| Anxiolytic medication^1^ | No (ref.) | 57/249 (23%) |  |  |  |
|  | Yes | 7/23 (30%) | 1.47 | 0.58-3.76 | 0.417 |
| Total AED load |  | 64/271 (24%) | 1.04 | 0.75-1.45 | 0.818 |
| Seizure severity^2^ |  | 64/272 (24%) | 1.03 | 1.00-1.07 | 0.044* |
| Status epilepticus^3^ | No (ref.) | 46/176 (26%) |  |  |  |
|  | Yes | 6/33 (18%) | 0.63 | 0.24-1.62 | 0.335 |
| Age |  | 64/272 (24%) | 0.98 | 0.96-1.00 | 0.075* |
| Sex | Female (ref.) | 32/113 (28%) |  |  |  |
|  | Male | 32/159 (20%) | 0.64 | 0.36-1.12 | 0.118 |
| Time since diagnosis |  | 64/272 (24%) | 1.00 | 0.99-1.00 | 0.240 |
| Ethnicity | Caucasian (ref.) | 56/252 (22%) |  |  |  |
|  | Other | 6/12 (50%) | 3.50 | 1.09-11.28 | 0.036* |
| Level of education | Low (ref.) | 14/72 (19%) |  |  |  |
|  | Medium/ high | 50/200 (25%) | 1.38 | 0.71-2.69 | 0.342 |
| Marital status | Partner (ref.) | 52/222 (23%) |  |  |  |
|  | No partner | 12/50 (24%) | 1.03 | 0.50-2.12 | 0.931 |
| Employment status | Not incapacitated to work (ref.) | 43/199 (22%) |  |  |  |
|  | Incapacitated to work | 21/73 (29%) | 1.47 | 0.80-2.69 | 0.219 |
| Social support | Adequate (ref.) | 59/263 (22%) |  |  |  |
|  | Not adequate | 5/9 (56%) | 4.32 | 1.13-16.61 | 0.033* |
| History of mood disorder treatment^4^ | No (ref.) | 50/241 (21%) |  |  |  |
|  | Yes | 14/31 (45%) | 3.15 | 1.45-6.81 | 0.004* |
| Mood disorder in family^5^ | No (ref.) | 41/193 (21%) |  |  |  |
|  | Yes | 23/79 (29%) | 1.52 | 0.84-2.76 | 0.166 |
| Most recent tumour grade^6^ | Low (grade II, ref.) | 34/135 (25%) |  |  |  |
|  | High (grade III & IV) | 30/137 (22%) | 0.83 | 0.48-1.46 | 0.523 |
| Extent of last resection | Biopsy (ref.) | 9/37 (24%) |  |  |  |
|  | Resection | 53/228 (23%) | 0.94 | 0.42-2.12 | 0.886 |
| Radiotherapy | No (ref.) | 13/55 (24%) |  |  |  |
|  | Yes | 51/217 (24%) | 0.99 | 0.50-2.00 | 0.983 |
| Chemo- and or immunotherapy | No (ref.) | 22/79 (28%) |  |  |  |
|  | Yes | 42/193 (22%) | 0.72 | 0.40-1.31 | 0.284 |
| Tumour lobe | Non-frontal (ref.) | 24/110 (22%) |  |  |  |
|  | Frontal | 40/162 (25%) | 1.18 | 0.66-2.09 | 0.584 |
| KPS | ≥70 (ref.) | 62/266 (23%) |  |  |  |
|  | <70 | 2/6 (33%) | 1.65 | 0.29-9.20 | 0.571 |

^1^Excluding anxiolytic medication for treatment of anxiety first prescribed after glioma diagnosis; ^2^Score 0-100 measured with the Liverpool Seizure Severity Scale; ^3^Status epilepticus was defined as ongoing seizures for ≥30 minutes if convulsive or ≥60 minutes if non-convulsive, because it has been thought long-term consequences might occur after this timeframe; ^4^Prior to glioma diagnosis, treatment started after glioma diagnosis was considered potentially in the causal pathway, as most medications with depressive and anxiety adverse effects were started after glioma diagnosis, and therefore not further analysed; ^5^First and/ or second degree relatives; ^6^Diffuse astrocytoma isocitrate dehydrogenase (IDH)-wildtype was considered high-grade; **p*<0.1; AAEs=Anxiety Adverse Effects; AEDs=Antiepileptic Drugs; CI=Confidence Interval; HADS-A=Hospital Anxiety and Depression Scale-Anxiety subscale; KPS=Karnofsky Performance Status; LEV=levetiracetam; ref.=reference category; uOR=unadjusted Odds Ratio; VPA=valproic acid

**Supplementary Table 6.** Univariable analyses of possible confounding predictor variables of subjective cognitive impairment

|  |  | **Cognitive impairment (≥2SD below the mean of normative data from the MOS)** | | | |
| --- | --- | --- | --- | --- | --- |
| Parameter |  | No. (%) | uOR | 95% CI | p-value |
| Current AED use, dichotomised | No AEDs | 14/88 (16%) |  |  |  |
|  | ≥1 | 38/184 (21%) | 1.38 | 0.70-2.70 | 0.353 |
| Current AED use, specified | Monotherapy VPA (ref.) | 9/32 (28%) |  |  |  |
|  | Monotherapy LEV | 12/85 (14%) | 0.42 | 0.16-1.12 | 0.084* |
|  | Other | 17/67 (25%) | 0.87 | 0.34-2.24 | 0.771 |
|  | No AEDs | 14/88 (16%) | 0.48 | 0.19-1.26 | 0.138 |
| Medications >1% risk of CAEs, excluding AEDs | No (ref.) | 40/235 (17%) |  |  |  |
|  | Yes | 12/37 (32%) | 2.34 | 1.09-5.04 | 0.030* |
| Total AED load |  | 51/271 (19%) | 1.36 | 0.98-1.91 | 0.070* |
| Seizure severity^1^ |  | 52/272 (19%) | 1.04 | 1.01-1.08 | 0.012* |
| Status epilepticus^2^ | No (ref.) | 34/176 (19%) |  |  |  |
|  | Yes | 7/33 (21%) | 1.12 | 0.45-2.81 | 0.802 |
| Age |  | 52/272 (19%) | 1.02 | 0.99-1.05 | 0.153 |
| Sex | Female (ref.) | 27/113 (24%) |  |  |  |
|  | Male | 25/159 (16%) | 0.59 | 0.32-1.09 | 0.093* |
| Time since diagnosis |  | 52/272 (19%) | 1.00 | 1.00-1.00 | 0.915 |
| Ethnicity^3^ | Caucasian (ref.) | 51/252 (20%) |  |  |  |
|  | Other | 1/13 (8%) | 0.33 | 0.04-2.59 | 0.290 |
| Level of education | Low (ref.) | 15/72 (21%) |  |  |  |
|  | Medium/ high | 37/200 (19%) | 0.86 | 0.44-1.69 | 0.666 |
| Marital status | Partner (ref.) | 44/222 (20%) |  |  |  |
|  | No partner | 8/50 (16%) | 0.77 | 0.34-1.76 | 0.536 |
| Employment status^4^ | Not incapacitated to work (ref.) | 30/199 (15%) |  |  |  |
|  | Incapacitated to work | 22/73 (30%) | 2.43 | 1.29-4.58 | 0.006* |
| Social support | Adequate (ref.) | 4/9 (44%) |  |  |  |
|  | Not adequate | 48/263 (18%) | 3.58 | 0.93-13.84 | 0.064* |
| History of mood disorder treatment^5^ | No (ref.) | 44/241 (18%) |  |  |  |
|  | Yes | 8/31 (26%) | 1.56 | 0.65-3.71 | 0.317 |
| Mood disorder in family^6^ | No (ref.) | 31/193 (16%) |  |  |  |
|  | Yes | 21/79 (27%) | 1.89 | 1.01-3.55 | 0.047* |
| Most recent tumour grade^7^ | Low (grade II, ref.) | 24/135 (18%) |  |  |  |
|  | High (grade III & IV) | 28/137 (20%) | 1.19 | 0.65-2.18 | 0.577 |
| Extent of last resection | Biopsy (ref.) | 5/37 (14%) |  |  |  |
|  | Resection | 47/228 (21%) | 1.66 | 0.61-4.50 | 0.317 |
| Radiotherapy | No (ref.) | 8/55 (15%) |  |  |  |
|  | Yes | 44/217 (20%) | 1.49 | 0.66-3.39 | 0.337 |
| Chemo- and or immunotherapy | No (ref.) | 16/79 (20%) |  |  |  |
|  | Yes | 36/193 (19%) | 0.90 | 0.47-1.74 | 0.761 |
| Tumour lobe | Non-frontal (ref.) | 22/110 (20%) |  |  |  |
|  | Frontal | 30/162 (19%) | 0.91 | 0.49-1.68 | 0.909 |
| KPS^8^ | ≥70 (ref.) | 48/266 (18%) |  |  |  |
|  | <70 | 4/6 (67%) | 9.08 | 1.62-51.03 | 0.012* |

^1^Score 0-100 measured with the Liverpool Seizure Severity Scale; ^2^Status epilepticus was defined as ongoing seizures for ≥30 minutes if convulsive or ≥60 minutes if non-convulsive, because it has been thought long-term consequences might occur after this timeframe; ^3^Regarding ethnicity we imputed a non-Caucasian for a missing variable to handle the problem of 0 in a cell; ^4^Employment status was not considered a confounding predictor variable, as being incapacitated to work is likely a cause of cognitive impairment; ^5^Prior to glioma diagnosis, treatment started after glioma diagnosis was considered potentially in the causal pathway, as most medications with depressive and anxiety adverse effects were started after glioma diagnosis, and therefore not further analysed; ^6^First and/ or second degree relatives; ^7^Diffuse astrocytoma isocitrate dehydrogenase (IDH)-wildtype was considered high-grade; ^8^KPS was not considered a confounding predictor variable, as a low KPS is likely a cause of cognitive impairment; **p*<0.1; AEDs=Antiepileptic Drugs; CAEs=Cognitive Adverse Effects; CI=Confidence Interval; KPS=Karnofsky Performance Status; LEV=levetiracetam; MOS=Medical Outcomes Study; ref.=reference category; uOR=unadjusted Odds Ratio; VPA=Valproic acid

**Supplementary Table 7.** Unadjusted and adjusted odds ratios of the predictor variables of depression with current AED use specified in the multivariable analysis

|  |  | **Depression (≥8 points on the HADS-D)** | | | | | |
| --- | --- | --- | --- | --- | --- | --- | --- |
| Parameter |  | uOR | 95% CI | p-value | aOR | 95% CI | p-value |
| Current AED use, specified | Monotherapy LEV (ref.) |  |  |  |  |  |  |
|  | Monotherapy VPA | 0.80 | 0.29-2.23 | 0.672 | 0.76 | 0.26-2.23 | 0.616 |
|  | Other | 0.84 | 0.38-1.85 | 0.658 | 0.61 | 0.25-1.47 | 0.270 |
|  | No AEDs | 0.40 | 0.17-0.93 | 0.065 | 0.42 | 0.17-1.06 | 0.065 |
| Medications >1% risk of DAEs^1^ | None (ref.) |  |  |  |  |  |  |
|  | ≥1 | 2.18 | 1.14-4.19 | 0.019* | 2.31 | 1.13-4.71 | 0.021* |
| Seizure severity |  | 1.03 | 1.00-1.07 | 0.055 | 1.02 | 0.99-1.06 | 0.208 |
| Level of education | Low (ref.) |  |  |  |  |  |  |
|  | Medium/ high | 2.84 | 1.15-7.00 | 0.024* | 2.18 | 0.85-5.61 | 0.105 |
| Employment status | Not incapicitated to work (ref.) |  |  |  |  |  |  |
|  | Incap. to work | 2.15 | 1.11-4.15 | 0.023* | 2.20 | 1.07-4.55 | 0.033* |
| Most recent tumour grade^2^ | Low (grade II, ref.) |  |  |  |  |  |  |
|  | High (grade III & IV) | 0.50 | 0.26-0.95 | 0.034* | 0.50 | 0.24-1.01 | 0.053 |
| KPS | ≥70 (ref.) |  |  |  |  |  |  |
|  | <70 | 10.37 | 1.84-58.42 | 0.008* | 9.42 | 1.55-57.15 | 0.015* |

^1^Excluding AEDs; ^2^Diffuse astrocytoma isocitrate dehydrogenase (IDH)-wildtype was considered high-grade; **p*<0.05; AED=Antiepileptic Drug; aOR=adjusted Odds Ratio; CI=Confidence Interval; DAEs=Depressive Adverse Effects; HADS-D=Hospital Anxiety and Depression Scale-Depression subscale; KPS=Karnofsky Performance Status; LEV=Levetiracetam; ref.=reference category; uOR=unadjusted Odds Ratio; VPA=Valproic acid

**Supplementary Table 8.** Unadjusted and adjusted odds ratios of the predictor variables of anxiety with current AED use specified in the multivariable analysis

|  |  | **Anxiety (≥8 points on the HADS-A)** | | | | | |
| --- | --- | --- | --- | --- | --- | --- | --- |
| Parameter |  | uOR | 95% CI | p-value | aOR | 95% CI | p-value |
| Current AED use, specified | Monotherapy LEV (ref.) |  |  |  |  |  |  |
|  | Monotherapy VPA | 0.40 | 0.14-1.15 | 0.088 | 0.55 | 0.19-1.65 | 0.289 |
|  | Other | 0.62 | 0.30-1.29 | 0.201 | 0.57 | 0.26-1.24 | 0.154 |
|  | No AEDs | 0.51 | 0.26-1.04 | 0.062 | 0.65 | 0.30-1.37 | 0.253 |
| Seizure severity |  | 1.03 | 1.00-1.07 | 0.044* | 1.03 | 1.00-1.07 | 0.087 |
| Age |  | 0.98 | 0.96-1.00 | 0.075 | 0.98 | 0.96-1.01 | 0.186 |
| Ethnicity | Caucasian (ref.) |  |  |  |  |  |  |
|  | Other | 3.50 | 1.09-11.28 | 0.036* | 2.85 | 0.82-9.89 | 0.098 |
| Social support | Adequate (ref.) |  |  |  |  |  |  |
|  | Not adequate | 4.32 | 1.13-16.61 | 0.033* | 3.65 | 0.82-16.13 | 0.088 |
| History of mood disorder treatment^1^ | No (ref.) |  |  |  |  |  |  |
|  | Yes | 3.15 | 1.45-6.81 | 0.004* | 2.86 | 1.26-6.50 | 0.012* |

^1^Prior to glioma diagnosis; **p*<0.05; AED=Antiepileptic Drug; aOR=adjusted odds ratio; CI=Confidence Interval; HADS-A=Hospital Anxiety and Depression Scale-Anxiety subscale; uOR=unadjusted Odds Ratio; LEV=Levetiracetam; ref.=reference category; VPA=Valproic acid

**Supplementary Table 9.** Unadjusted and adjusted odds ratios of the predictor variables of subjective cognitive impairment with current AED use specified in the multivariable analysis

|  |  | **Impaired subjective cognition (≥2SD below the mean of normative data from the MOS)** | | | | | |
| --- | --- | --- | --- | --- | --- | --- | --- |
| Parameter |  | uOR | 95% CI | p-value | aOR | 95% CI | p-value |
| Current AED use, specified | Monotherapy VPA (ref.) |  |  |  |  |  |  |
|  | Monotherapy LEV | 0.42 | 0.16-1.12 | 0.084 | 0.40 | 0.14-1.11 | 0.078 |
|  | Other AED use | 0.87 | 0.34-2.24 | 0.771 | 0.48 | 0.15-1.51 | 0.209 |
|  | No AEDs | 0.48 | 0.19-1.26 | 0.138 | 0.67 | 0.22-1.98 | 0.463 |
| Medications >1% risk of CAEs | None (ref.) |  |  |  |  |  |  |
|  | ≥1 | 2.34 | 1.09-5.04 | 0.030* | 2.11 | 0.93-4.78 | 0.075 |
| Seizure severity |  | 1.04 | 1.01-1.08 | 0.012* | 1.04 | 1.00-1.08 | 0.030* |
| Total AED load |  | 1.36 | 0.98-1.91 | 0.070 | 1.37 | 0.79-2.39 | 0.264 |
| Sex | Female (ref.) |  |  |  |  |  |  |
|  | Male | 0.59 | 0.32-1.09 | 0.093 | 0.62 | 0.32-1.18 | 0.143 |
| Social support | Adequate (ref.) |  |  |  |  |  |  |
|  | Not adequate | 3.58 | 0.93-13.84 | 0.064 | 2.80 | 0.60-13.06 | 0.190 |
| Mood disorder in family^1^ | No (ref.) |  |  |  |  |  |  |
|  | Yes | 1.89 | 1.01-3.55 | 0.047* | 1.48 | 0.74-2.93 | 0.265 |

^1^Excluding AEDs; ^2^First and/ or second degree relatives; **p*<0.05; AED=Antiepileptic Drug; aOR=adjusted Odds Ratio; CAEs=Cognitive Adverse Effects; CI=Confidence Interval; LEV=Levetiracetam; MOS=Medical Outcomes Study; ref.=reference category; uOR=unadjusted Odds Ratio; VPA=Valproic acid

**References**

1. Klein M, Heimans JJ, Aaronson NK, van der Ploeg HM, Grit J, Muller M, et al. Effect of radiotherapy and other treatment-related factors on mid-term to long-term cognitive sequelae in low-grade gliomas: a comparative study. Lancet. 2002;360(9343):1361-8.

2. Klein M, Engelberts NH, van der Ploeg HM, Kasteleijn-Nolst Trenite DG, Aaronson NK, Taphoorn MJ, et al. Epilepsy in low-grade gliomas: the impact on cognitive function and quality of life. Annals of neurology. 2003;54(4):514-20.

3. Scott-Lennox J, Bryant-Comstock L, Lennox R, Baker GA. Reliability, validity and responsiveness of a revised scoring system for the Liverpool Seizure Severity Scale. Epilepsy research. 2001;44(1):53-63.

4. Zigmond AS, Snaith RP. The hospital anxiety and depression scale. Acta psychiatrica Scandinavica. 1983;67(6):361-70.

5. Rooney AG, McNamara S, Mackinnon M, Fraser M, Rampling R, Carson A, et al. Screening for major depressive disorder in adults with cerebral glioma: an initial validation of 3 self-report instruments. Neuro-oncology. 2013;15(1):122-9.

6. Bell ML, Fairclough DL, Fiero MH, Butow PN. Handling missing items in the Hospital Anxiety and Depression Scale (HADS): a simulation study. BMC Res Notes. 2016;9(1):479.

7. Hays RD, Sherbourne CD, Mazel R. User's Manual for the Medical Outcomes Study (MOS) Core Measures of Health-Related Quality of Life. . Santa Monica, CA: RAND Corporation; 1995.

8. Bosma I, Reijneveld JC, Douw L, Vos MJ, Postma TJ, Aaronson NK, et al. Health-related quality of life of long-term high-grade glioma survivors. Neuro-oncology. 2009;11(1):51-8.
